# One-year impact of COVID-19 pandemic on renal replacement therapy and kidney transplantation in a tertiary center in Southern Brazil

**DOI:** 10.1590/2175-8239-JBN-2022-0034en

**Published:** 2022-09-12

**Authors:** Pedro do Valle Teichmann, Marina Oliboni Moschetta, Rodrigo Fontanive Franco, Alessandra Rosa Vicari, Gérson Luiz da Silva Nunes, Maria Angela Kalil Nader Lazzaretti, Miriam Gressler Gomes, Silvia Maria Dorigoni, Paulo Roberto Dall’Agnol, Adriana Klafke, Fernando Saldanha Thomé, Fabio Spuldaro, Homero Agra, Rafael de Almeida, Darlan Martins Lara, Andrea Carla Bauer, Cristina Karhol, Roberto Ceratti Manfro

**Affiliations:** 1 Hospital de Clínicas de Porto Alegre, Divisão de Nefrologia, Porto Alegre, RS, Brazil.; 2 Hospital de Clínicas de Porto Alegre, Divisão de Transplante, Porto Alegre, RS, Brazil.; 3 Universidade Federal do Rio Grande do Sul, Faculdade de Medicina, Porto Alegre, RS, Brazil.; 4 Clínica de Hemodiálise Campo Bom, Campo Bom, RS, Brazil.; 5 Clínica do Rim de Alvorada, Alvorada, RS, Brazil.; 6 Centro Nefrológico de Taquara, Taquara, RS, Brazil.; 7 Clínica de Hemodiálise de São Leopoldo, São Leopoldo, RS, Brazil.; 8 Fundação Hospitalar Santa Terezinha, Clínica Renal Erechim, Erechim, RS, Brazil.; 9 Hospital São Vicente de Paulo, Hemodiálise Osório, Osório, RS, Brazil.; 10 Instituto de Doenças Renais, Porto Alegre, RS, Brazil.; 11 Centro de Dialise e Transplante, Porto Alegre, RS, Brazil.; 12 UNI-RIM, Clínica de Doenças Renais, Santa Cruz, RS, Brazil.; 13 Serviço de Nefrologia, Santa Casa de Pelotas, RS, Brazil.; 14 Hospital de Caridade de Carazinho, Centro de Nefrologia, Carazinho, RS, Brazil.; 15 Centro de Prevenção e Tratamento de Doenças Renais, Novo Hamburgo, RS, Brazil.

**Keywords:** Kidney Transplantation, SARS-CoV-2, COVID-19, Renal Replacement Therapy, Kidney Transplantation, SARS-CoV-2, COVID-19, Renal Replacement Therapy

## Abstract

**Introduction:**

Patients on renal replacement therapy (RRT) and kidney transplant recipients (KTR) present multiple factors that may increase the risk of death from coronavirus disease 2019 (COVID-19). This study aimed to evaluate the incidence and impact of COVID-19 in RRT patients and KTRs.

**Methods:**

Between March 2020 and February 2021, we monitored the RRT population of thirteen dialysis facilities that refer patients for transplantation to our center, a tertiary hospital in Southern Brazil. In the same period, we also monitor COVID-19 incidence and mortality in our KTR population. Demographical, clinical, and COVID-19-related information were analyzed.

**Results:**

We evaluated 1545 patients in the dialysis centers, of which 267 (17.4%) were infected by COVID-19 and 53 (19.9%) died. Among 275 patients on the kidney transplant waiting list, 63 patients (22.9%) were infected and seven (11.1%) died. COVID-19 was the leading cause of death (29.2%) among patients on the waiting list. Within the population of 1360 KTR, 134 (9.85%) were diagnosed with COVID-19 and 20 (14.9%) died. The number of kidney transplants decreased by 56.7% compared with the same period in the previous twelve months.

**Conclusion:**

In the study period, patients on RRT and KTRs presented a high incidence of COVID-19 and high COVID-19-related lethality. The impact on the patients on the transplant waiting list was less pronounced. The lethality rate observed in both cohorts seems to be related to age, comorbidities, and disease severity.

## Resumo

**Introdução:**

Pacientes em terapia renal substitutiva (TRS) e receptores de transplante renal (RTR) apresentam múltiplos fatores que podem aumentar o risco de óbito por doença do coronavírus 2019 (COVID-19). Este estudo teve como objetivo avaliar incidência e impacto da COVID-19 em pacientes em TRS e RTR.

**Métodos:**

Entre Março de 2020 e Fevereiro de 2021, monitoramos a população em TRS de treze unidades de diálise que encaminham pacientes para transplante ao nosso centro, um hospital terciário no Sul do Brasil. No mesmo período, também monitoramos a incidência e mortalidade da COVID-19 em nossa população de RTR. Foram analisadas informações demográficas, clínicas e relacionadas à COVID-19.

**Resultados:**

Avaliamos 1545 pacientes nos centros de diálise, dos quais 267 (17,4%) foram infectados pela COVID-19 e 53 (19,9%) foram a óbito. Entre 275 pacientes na lista de espera para transplante renal, 63 (22,9%) foram infectados e sete (11,1%) foram a óbito. COVID-19 foi a principal causa de óbito (29,2%) entre pacientes na lista de espera. Dentre a população de 1360 RTR, 134 (9,85%) foram diagnosticados com COVID-19 e 20 (14,9%) foram a óbito. O número de transplantes renais diminuiu em 56,7% comparado ao mesmo período nos doze meses anteriores.

**Conclusão:**

No período do estudo, pacientes em TRS e RTR apresentaram alta incidência de COVID-19 e alta letalidade relacionada à COVID-19. O impacto nos pacientes na lista de espera para transplante foi menos pronunciado. A taxa de letalidade observada em ambas as coortes parece estar relacionada à idade, comorbidades e gravidade da doença.

## Introduction

The first COVID-19 case was reported by the Chinese government in late December 2019^1^. In February 2020, the World Health Organization (WHO) acknowledged the disease caused by the SARS-CoV-2 virus as a pandemic. In Brazil, the first case was registered on February 26, 2020, in the state of Rio Grande do Sul (RS) on February 29, and the first reported death in that state occurred in March 2020^2^. In most Brazilian states, including RS, COVID-19 reached alarming numbers, leading to a severe public health crisis^3^. In one year, there were 932,808 covid cases and 13,045 deaths in RS state^4^.

Patients with chronic kidney diseases (CKD) on renal replacement therapy (RRT) tend to be older and present several comorbidities. They are exposed to health care environments (RRT hospitals and clinics), and usually need public or shared forms of transportation to attend the hemodialysis sessions^5^. These factors increase exposure and pose a challenge in maintaining social distancing. Also, kidney transplant recipients (KTR) are a high-risk population for COVID19 complications and death^6,7^, mainly due to many comorbidities such as hypertension, diabetes, cardiac conditions, and the necessary state of immunosuppression and also because they are affected by metabolic and inflammatory conditions^6,8^.

In the present study, we evaluated the one-year impact of the SARS-CoV-2 pandemic in RRT and kidney transplantation in a tertiary center located in the state of Rio Grande do Sul, Brazil, that became a reference center for the care of severe cases of COVID-19 patients.

## Patients and Methods

### Patients in Renal Replacement Therapy

Clinics and hospitals with dialysis facilities associated with the kidney transplantation program of the *Hospital de Clínicas de Porto Alegre* (HCPA) received a structured questionnaire about general and COVID-19-related information between March 2020 and February 2021. The following characteristics were sought: (a) demographic: age, sex, race; (b) CKD-RRT associated characteristics: CKD etiology, type of RRT (hemodialysis [HD] or peritoneal dialysis [PD]); time on RRT; presence of comorbidities (hypertension, diabetes, heart, lung and vascular brain diseases, hepatitis B, hepatitis C, hepatic cirrhosis, and HIV infection), and transplant wait-list status (active or non-active); and (c) COVID-19 related information: confirmatory test (PCR, antigen, serology), disease severity, outcome (clinical improvement without hospital admission, hospital admission with discharge, ICU admission, death). Only symptomatic patients with a positive COVID-19 test were included in the study and the main outcomes were disease incidence and mortality by COVID-19. Disease severity was classified as mild in patients with symptoms who did not require hospital admission, moderate in patients who did require hospital admission, and severe in patients admitted to the intensive care unit (ICU). Patients were followed throughout the study period up to the end of April 2021.

### Kidney Transplant Recipients

We evaluated COVID-19 infections and incidence in our population of KTRs. The following characteristics were sought: (a) demographic: age, sex, race, CKD etiology; (b) kidney transplantation-associated characteristics: donor type, transplant time, immunosuppressive therapy, serum creatinine at baseline and at COVID-19 diagnosis, comorbidities; and (c) COVID-19 related information: confirmatory test (PCR, antigen, serology), disease severity, time from transplant to infection, modification of the immunosuppressive regimen, main outcomes (clinical improvement without hospital admission, hospital admission with discharge, ICU admission, death), secondary outcomes (acute kidney injury with or without need for RRT, graft loss of function) and disease management.

Symptomatic KTRs with clinical suspicion of COVID-19 infection were attended at HCPA or in other facilities under the guidance of the transplant nephrologists of the HCPA team. The population at risk included all KTRs followed at the institution in the study period. Patients were followed throughout the study period up to the end of April 2021.

### Statistical Analysis

Data is presented in absolute numbers, percentages, and frequencies. In the univariate analysis, continuous variables are presented as mean ± standard deviation or as median and interquartile range according to data distribution. Categorical variables are presented as frequencies. The Poisson regression with robust estimator was used to control for confounding factors. Variables with p-value ≤0.10 on univariate analysis were entered into the multivariate model. Variables included in the risk of death assessment were: (a) patients in RRT: age, ethnicity, gender, dialysis type (hemodialysis or peritoneal dialysis), presence of hypertension, diabetes mellitus, ischemic heart disease, cardiac failure, history of stroke, hepatitis C infection, HIV infection, liver cirrhosis, chronic lung disease, time in RRT, status in the kidney transplant waiting list (listed versus not listed), and COVID19 severity; (b) KTR: age, ethnicity, gender, CKD etiology, BMI, presence of hypertension, diabetes mellitus, cardiovascular disease, autoimmune disorders, cancer, liver disease, chronic lung disease, transplant time, donor type (deceased versus living) immunosuppressive therapy, graft function, and COVID-19 severity. A P-value lower than 0.05 was considered statistically significant. Statistical analysis was performed using SPSS version 18.0 software (SPSS, Inc., Chicago, IL, USA).

The study was approved by the Institutional Review Board of the HCPA, Porto Alegre/RS, Brazil (Ref. No. 2021–0078), which was registered online (www.saude.gov.br/plataformabrasil: CAAE Ref. No. 44299821.5.0000.5327).

## Results

### Renal Replacement Therapy Cohort

We invited sixteen dialysis facilities associated with the HCPA kidney transplant center to participate, and thirteen (81.3%) reported their data. In the study period, the number of patients in RRT in these facilities was 1545, and 322 deaths by any cause occurred, representing a crude mortality rate of 20.8%. Two hundred and sixty-seven cases of COVID-19 occurred (infection rate: 17.3%). The most frequent diagnosis method was RT-PCT (228 patients; 85.4%), followed by serology (24 patients; 9.0%) and antigen test (14 patients; 5.2%). Fifty-three deaths occurred among the COVID-19 infected patients representing a 19.9% lethality rate and a mortality rate of 3.43%. Among the patients in RRT, COVID-19 infection directly caused 16.5% of all deaths in the study period. No deaths occurred in the 195 cases of mild or moderate severity, and 53 among the 72 patients with severe illness died, a lethality rate of 73.6%.

Table 1 shows the demographic and clinical characteristics of COVID-19 in RRT patients. Univariate comparison of survivors and non-survivors showed that deceased patients were older, mostly male, and presented a higher prevalence of cardiovascular diseases. Patients in the kidney transplant waiting list were less likely to die due to COVID-19. Table 2 shows the odds ratio (OR) for death in the univariate analysis (age, sex, cardiovascular conditions, transplant waiting list, and disease severity), and in the multivariate analyses, age, cardiovascular conditions, and disease severity remained independent risk factors for death.

**Table 1.** Demographic and clinical characteristics of patients on renal replacement therapy diagnosed with COVID-19 between march 2020 and february 2021

	Total (N = 267)	Deaths (n = 53)	Survivors (n = 214)	P
Age (years; median P25–75)	58.00 (45.0; 70.5)	66.0 (57.5; 73.5)	55.0 (43.0; 69.0)	**<0.001**
Ethnicity (Caucasian/Non-Caucasian)	209/58	43/10	166/48	0.932
Sex (male/female)	152/115	39/14	113/101	**0.010**
Dialysis modality (HD/PD)	262/5	53/0	209/5	0.261
Transplant waiting list (yes)	32 (12%)	1 (1.8%)	31 (14.5%)	**0.008**
High blood pressure (yes)	229 (85.8 %)	48 (90.6%)	181 (84.6%)	0.264
Diabetes (yes)	107 (40.1%)	27 (50.9%)	80 (37.4%)	0.071
Cardiovascular disease (yes)	80 (30.0%)	30 (56.6%)	50 (23.4%)	**<0.001**
Previous stroke (yes)	24 (9.0%)	8 (15.1%)	16 (7.5%)	0.083
Liver disease (yes)	23 (8.6%)	7 (13.2%)	16 (7.5%)	0.183
HIV infected (yes)	6 (2.2%)	2 (3.8%)	4 (1.9%)	0.402
CPOD (yes)	13 (4.9%)	4 (7.5%)	9 (4.2%)	0.297
Time in RRT (months; median P25–75)	28.5 (10.0; 61.0)	26.0 (8.25; 63.0)	29.0 (10.0; 60.0)	0.955
COVID19 severity (mild/moderate/severe)				
Mild	136 (50.9%)	0 (0.0%)	136 (63.5%)	**<0.001**
Moderate	59 (22.1%)	0 (0.0%)	59 (27.5%)	**<0.001**
Severe	72 (27.0%)	53 (100%)	19 (8.8%)	**<0.001**

HD: hemodialysis; PD: peritoneal dialysis; CPOD: chronic obstructive pulmonary disease; RRT: renal replacement therapy.

**Table 2.** Analysis of risk factors for death by COVID-19 in patients on renal replacement therapy

Variable	OR (95% CI)	P
**Univariate Analysis**		
Age (years)	1.007 (1.004 – 1.009)	**<0.001**
Sex (male)	1.668 (1.085 – 2.565)	**0.020**
Transplant waiting list (yes)	0.511 (0.323 – 0.808)	**0.040**
Cardiovascular disease (yes)	1.315 (0.971 – 1.781)	**<0.001**
Diabetes (yes)	0.956 (0.599 – 1.526)	0.094
ICU admission (yes)	84.816 (12.14 – 592.32)	**<0.001**
**Multivariate Analysis**		
Age (years)	1.008 (1.009 – 1.037)	**0.02**
Cardiovascular disease (yes)	2.369 (1.458 – 3.900)	**<0.001**
ICU admission (yes)	93.161 (14.03 – 618.58)	**<0.001**

In the study period, the kidney transplant waiting list had 275 patients. Sixty-three (22.9%) were diagnosed with COVID-19. Twenty-four deaths by any cause occurred among these patients, representing a crude mortality rate of 8.73%. Seven deaths occurred due to COVD-19 representing an 11.1% lethality rate. Therefore, 29.2% of the deaths of patents on the waiting list were due to COVID-19, which was the main cause of death.

**Figure 1.**
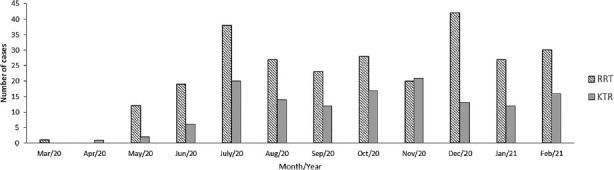
Number of COVID-19 new cases among patients on renal replacement therapy and kidney transplant recipients between March 2020 and February 2021.

**Figure 2.**
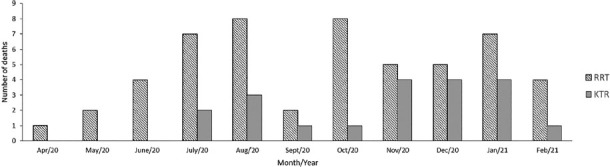
Deaths by COVID-19 among patients on renal replacement therapy and kidney transplant recipients diagnosed between March 2020 and February 2021.

Figure 1 shows the monthly frequency of COVID-19 cases among patients on RRT and KTR in the study period. Figure 2 displays the monthly number of deaths in this period in the same cohorts.

### Kidney Transplant Cohort

Throughout the study period, 1360 KTR were at risk of contracting COVID-19, of whom 134 were infected, representing a one-year incidence of 9.85%. The diagnostic methods were RT-PCR in 126 patients (96.9 %), followed by serology in 3 patients (2.3 %) and antigen test in 1 patient (0.7%). Twenty deaths by COVID-19 occurred, a fatality rate of 14.9 %. No deaths occurred in the 92 cases considered of mild or moderate severity, and the 20 casualties occurred among the 43 patients with severe illness, a mortality rate of 46.5%.

Table 3 shows the demographic and clinical characteristics of COVID-19-infected KTR and a univariate comparison of survivors and non-survivors. All patients who died had severe disease, and these recipients also had a higher frequency of liver disease and a borderline higher frequency of previous malignancy. Table 4 shows the multivariate analysis where only disease severity (ICU admission) remained significantly associated with death.

**Table 3.** Demographic and clinical characteristics of kidney transplant recipients diagnosed with COVID-19 between march 2020 and february 2021

	Total (N = 134)	Death (n = 20)	Survivor n = 114)	P
Age (years; median P25–75)	54.00 (43.0; 62.0)	57.0 (47.5; 66.0)	53.0 (42.0; 62.0)	0.213
Ethnicity (Caucasian/non-Caucasian)	116/18	18/2	98/16	0.745
Sex (male/female)	71/63	9/11	62/52	0.438
BMI (kg/m2; median P25–75)	27.85 (24.6;31.3)	27.5 (24.5;31.5)	27.8 (24.6;31.5)	0.988
Donor type (living/deceased)	17/117	1/19	16/98	0.263
High blood pressure (yes)	116 (86.6 %)	17 (85.0%)	99 (86.8%)	0.824
Diabetes (yes)	80 (40.3%)	10 (50.0%)	44 (38.6%)	0.341
Autoimmune disease (yes)	6 (4.5%)	2 (10.0%)	4 (3.5%)	0.247
Cardiovascular disease (yes)	25 (18.7%)	2 (10.0%)	23 (20.2%)	0.251
Cancer (yes)	9 (6.7%)	4 (20.0%)	5 (4.4%)	**<0.01**
Liver disease (yes)	13 (9.7%)	6 (30.0%)	7 (6.1%)	**<0.001**
Baseline serum creatinine (mg/dL)	1.5 (1.0;2.08)	1.4 (1.2; 2.3)	1.5 (1.00; 2.07)	0.407
Transplant months at COVID diagnosis (median P25–75)	68.0 (28.0; 107.0)	67.5 (37.2; 114.0)	69.0 (28.0; 106.0)	0.780
Need for mechanical ventilation	27 (20.1%)	18 (90.0%)	16 (14.0%)	**<0.001**
COVID-19 severity				
Mild	26 (19.4%)	0 (0.0%)	26 (22.8%)	**<0.001**
Moderate	66 (49.2%)	0 (0.0%)	66 (57.9%)	**<0.001**
Severe	42 (31.3%)	20 (100.0%)	22 (19.2%)	**<0.001**

BMI: body mass index.

**Table 4.** Analysis of risk factors for death by COVID-19 in kidney transplant recipients

Variable	OR (95% CI)	P
**Univariate Analysis**		
Cancer (yes)	1.196 (0.928 – 1.796)	0.118
Liver disease (yes)	1.326 (1.127 – 1.855)	**0.007**
ICU admission (yes)	1.865 (1.615 – 2.124)	**<0.001**
**Multivariate Analysis**	
ICU Admission (yes)	18.155 (5.778 – 56.874)	**<0.001**

Only fifty-two kidney transplants, all with deceased donors, occurred in the study period. The number of kidney transplants decreased by 56.7% compared to the previous 12 months when 120 transplants were performed.

## Discussion

Our study shows the one-year impact of COVID-19 infection in patients on RRT and KTR in the practice of a tertiary hospital that was prioritized to care for severe infection cases in its region. Our main findings were a high incidence of disease and fatality rate in KTR and patients on RRT. RRT patients on the transplant waiting list presented a lower fatality rate compared with unlisted RRT patients and KTRs.

A very large populational study in France revealed that patients with end-stage renal disease on dialysis and kidney transplantation are strongly associated with hospitalization and in-hospital mortality of COVID-19 infected individuals^8^. Chronic kidney disease was also a strong risk factor for hospital admission in a large North American cohort^9^.

Infection and mortality rates by COVID-19 in RRT vary substantially in the literature, perhaps due to differences in the studied populations^10,11,12^. In Brazil, Pio Abreu et al, in a national online survey of COVID-19 with 37,852 patients on RRT with hemodialysis, found a 3.4% infection rate and a 27.7% fatality rate^11^. Couchoud and collaborators, analyzed a large population from the French REIN registry and found an identical infection rate and a fatality rate of 21.2%^13^. Jager et al.^14^, analyzed the data of 36,135 patients receiving kidney replacement therapy in seven European countries in 2020 from the ERA-EDTA Registry and observed a mortality rate of 20%. Smaller single-center cohorts that collected data during COVID-19 outbreaks reported higher infection rates. A report from Wuhan, China, showed an infection incidence of 40% among 143 RRT patients^10^. One report from Turkey revealed an infection incidence of 54.7% in a small cohort of patients^15^. In the present study, the one-year infectivity rate reached 17.3%, which is much higher than that found in multicenter reports, perhaps due to the more extended period of the study. The fatality rate observed in our study is similar to the one found in the large-scale studies^11,12,13,14^.

In a very large North-American cohort, the identified risk factors for mortality in the RRT population were older age, heart disease, and markers of frailty^16^. Although our database did not capture frailty, the other risk factors were identical. Moreover, severe disease is an expected risk factor.

An interesting subpopulation is RRT patients actively listed for kidney transplantation. Patients on the waiting list for kidney transplant are usually healthier than the general population of patients on RRT. Therefore, they may be expected to be less impacted by the disease than patients that are not candidates for kidney transplantation, specifically with lower lethality rates. In our study, the observed COVID-19 mortality rate was substantially lower in waiting list patients than in the RRT population. Moreover, the fatality rate in waiting list RRT patients was also lower than the one observed in KTR, and this finding contrasts with a previous single-center study^17^. However, a much larger database study that linked data from the UK Transplant Registry with those from Public Health England and NHS Digital Tracing Services showed a 2.5 times higher mortality in transplant recipients compared with waitlisted patients^18^. Moreover, a propensity match score analysis in Brazilian populations of KTR and patients on RRT showed that both cohorts had increased 30-day mortality after COVID-19 diagnosis. KTR, despite lower death risk at baseline, presented an increased risk of death than dialysis patients^19^.

Almost one-tenth of our population of KTRs had COVID-19 during the first year of the pandemic. This frequency is almost 40% higher than the rate for the general population of the RS state in the same period, according to the state Secretary of Health^4^. We believe that the higher frequency in KTRs is due to the fact these patients are more frequently tested since they receive regular medical care and are more likely to have clinically relevant disease. All KTRs who died due to COVID-19 had severe forms of the disease, and among the patients with severe infection, the fatality rate was much higher. Variables related to death were older age, presence of previous liver disease, and severe COVID-19. However, only severe disease was associated with death on the multivariate model, perhaps because of a sample-size matter.

A review of the one-year impact of the COVID-19 pandemic in KTR showed a negative effect on allograft and patient survival and on all aspects of transplant care, including referrals and listing for transplantation, organ procurement, and donation, as well as increased waitlist mortality^20^. Likewise, another international study with the participation of Brazilian transplant centers reported that a significant impact on living-donor kidney transplantation also occurred in the first year of the pandemic^21^.

Also, in a large Brazilian multicenter study, the probability of death reached 21% in 90 days, and deaths were related to older age, longer time after transplantation, comorbidities (hypertension and cardiovascular disease), immunosuppression with tacrolimus, mycophenolate, recent high dose of steroids, and dyspnea as COVID-19 symptom^6^. However, a very large transplant program in Brazil reported that the adoption of sequential coordinated safety measures allowed the maintenance of the transplant program. Nevertheless, the elevated mortality observed in most centers and registries also occurred in the infected population of KRT followed in this center^22^.

Data from a United States national registry (United Network for Organ Sharing - UNOS) in 2020 involving more than 190.000 KTRs revealed that 16% of the deaths in this population occurred due to COVD-19. Mortality was associated with race/ethnicity, primary insurance payer, lower education level, and preemptive transplantation (protective), and in 2020, 908 all-cause additional deaths were observed compared to the previous year leading to a 20% increment of the overall mortality compared with 2019. The most prominent upward trend in KTRs mortality in 2020 occurred during the initial spring surge of the pandemic in that country^23^.

It is important to note that our study and the articles mentioned above were conducted before the widespread COVID-19 vaccination. Nevertheless, the effectiveness of vaccination in RRT and KTR populations differ substantially. While strong antibody-generating effects occur in the RRT population, antibody-generating responses to vaccination are much weaker in KTRs, possibly requiring multiple booster shots^24,25,26,27^.

This study demonstrated the impact of COVID-19 pandemic on RRT patients and KTR from southern Brazil. Our real-world data provided important observations for a population severely affected by the pandemic. However, this study also has some limitations. First, only patients with the symptomatic form of the disease were identified in both cohorts, since we did not screen asymptomatic individuals; second, we did not perform antibody assays before or after infection; and third, the vaccination status of the populations changed, and as the pandemic evolves, differences in the pathogenicity of new variants of concern could not be addressed.

## Conclusion

Our study demonstrated the one-year impact of COVID-19 infection in the local RRT and KTR populations. We confirmed elevated lethality rates in both populations with a relative sparing of RRT on patients in the kidney transplant waiting list. Our data support the utmost need for preventive measures, including distancing, effective masks, well-ventilated environments, and massive effective vaccination of the general and susceptible populations.
